# Postgraduate medical education in change: A long journey

**DOI:** 10.3205/zma001724

**Published:** 2024-11-15

**Authors:** Eva K. Hennel, Folkert Fehr, Marjo Wijnen-Meijer, Sigrid Harendza

**Affiliations:** 1 Schweizerisches Institut für ärztliche Weiter und Fortbildung SIWF, Bern, Switzerland; 2 Gemeinschaftspraxis für Kinder- und Jugendmedizin, Lehrpraxis der Ruprecht-Karls-Universität Heidelberg, Sinsheim an der Elsenz, Germany; 3 TUD Dresden University of Technology, Medical Faculty and University Hospital Carl Gustav Carus, Institute of Medical Education, Dresden, Germany; 4 Universitätsklinikum Hamburg-Eppendorf, III. Medizinische Klinik und Poliklinik, Hamburg, Germany

## Editorial

This special issue deals with residency training, which is referred to as “Weiterbildung” in Switzerland and Germany and “Ausbildung” in Austria. It shows examples how residency training can succeed and where there are still hurdles in implementation. A qualitative study by the European junior doctors in 2023 showed that many doctors in training, regardless of the country of their profession, are disappointed and dissatisfied with their work [1]. According to the study, this is influenced by three factors: the working environment, the quality of training and the work-life balance. These factors are interlinked. As medical teachers, we are responsible for the quality of residency training and can make a significant contribution to improving the overall situation.

The position paper “The future of graduate medical education in Germany” by the GMA-Committee on residency training from 2013 [2] already pointed out the lack of evidence in the educational implementation of training. It was found that data on competence definition, curriculum, implementation and quality assurance were missing. The last special issue “postgraduate education” of the GMS JME was published in 2017. The main focus was on the change towards competence-based residency training and the international experiences in this area were presented. Since then, competence-based training has spread, and the first successes, but also stumbling blocks, from the German-speaking world are becoming apparent. The aim of this special issue is to bring together contributions to innovative concepts, theories, teaching formats or educational programmes, especially from Germany, Austria and Switzerland, in order to identify solutions for formats that can be used in residency training. Our authors shed light on the latest developments and challenges. Most studies or projects have been developed against the background of current residency training in Germany or Switzerland.

The authors bring different experiences and areas of expertise to this special issue in form of research reports and project reports. This diversity enriches the discussion and opens up new horizons. Reforming residency training includes not only didactic but also structural and political aspects. Consideration is given to promotion of social legislation, the possibilities within the regulations on residency training and aspects of concrete curriculum development. Competence-based medical education (CBME) including the use of Entrustable Professional Activities is also addressed in several papers. Several articles deal with the situation of the trainees, for example with doctors’ health, the use of feedback and the choice of the specialty. Concrete examples of courses and teaching methods can be found in the various project reports. In addition, unresolved aspects of assessment are addressed as well as the exceptional perspective of general practice. 

With this special issue, we would like to re-initiate the discussion about the current challenges of residency training and provide suggestions for the development of new strategies in German-speaking countries. We interpret it as a positive sign that at many residency training sites new teaching formats are being established which promote the quality of residency training in an evidence-based way and can also help to answer the question of good assessment. In addition, findings from medical education also seem to find their way into the political discussion. We encourage our readers to contribute their own thoughts on the residency training topics. Discussions and comments are very welcome. Some of the topics presented in this issue have already been discussed in the committee on residency training or in our monthly webinar series “45 Minuten für die Weiterbildung” [https://gesellschaft-medizinische-ausbildung.org/ausschuesse/weiterbildung/id-45-minuten-fuer-die-weiterbildung.html]. We plan to continue this public discussion and continue to invite guests who contribute different points of view, such as that from teachers and trainees from both hospital and outpatient settings, of the State Medical Associations, the Federal Medical Association, and international bodies. We invite especially the residents to contribute their important perspective. 

The articles in this special issue provide the basis for a multifaceted discussion on the relevant topic of residency training. We hope that you will be inspired, can gain new insights, and enjoy reading.

## Authors’ ORCIDs

- Eva K. Hennel: [0000-0002-7625-5785]
- Folkert Fehr: [0000-0001-8495-4167]
- Marjo Wijnen-Meijer: [0009-0006-4861-8098]
- Sigrid Harendza: [0000-0002-7920-8431]

## Competing interests

The authors declare that they have no competing interests. 
